# Success Rate and Related Factors of Vitapex Pulpectomy in Primary Teeth: A Retrospective Study

**DOI:** 10.1055/s-0042-1758792

**Published:** 2023-06-05

**Authors:** Duangsamon Mekkriangkrai, Siriruk Nakornchai, Varangkanar Jirarattanasopha

**Affiliations:** 1 Department of Pediatric Dentistry, Faculty of Dentistry, Mahidol University, Bangkok, Thailand

**Keywords:** primary teeth, pulpectomy, related factors, success rate, Vitapex

## Abstract

**Objectives** Our aims were to evaluate the Vitapex pulpectomy (PE) success rate, Vitapex resorption rate, and their associated factors in primary teeth.

**Material and Methods** This retrospective study evaluated the clinical records of Vitapex PE-treated patients at the Pediatric Dental Clinic, Faculty of Dentistry, Mahidol University, from 2013 to 2019. The patient's and pulpectomized tooth's characteristics, procedure, materials used, and type of operator were recorded. A dentist evaluated and compared the periapical lesion, root status, obturation quality, and Vitapex resorption on preoperative, immediate, and follow-up digital radiographs. PE failure was defined as radiographic lesion progression.

**Statistical Analysis** The Kaplan–Meier method was used to estimate the Vitapex PE success rate and Vitapex resorption rate. Multivariate Cox regression was used to determine the related factors.

**Results** In total, 647 Vitapex PE teeth from 448 patients (19–121-month-old) were analyzed. The follow-up periods ranged from 6 to 60 months. The success rate was 88.9 and 68.1% at the 12- and 24-month follow-up, respectively, and remained stable at 53.8% at the 36 to 60-month follow-ups. The factors related to Vitapex PE failure were age and a preoperative pathologic lesion. More than 50% of the pulpectomized teeth presented Vitapex resorption faster than physiologic root resorption at the 12-month follow-up. The patients' age at treatment and the obturation quality were significantly related to the Vitapex resorption rate.

**Conclusions** The success rate of Vitapex PE decreased time dependently and was related to the patient's age at treatment and a preoperative lesion. The Vitapex resorption was faster than root resorption and was associated with the patient's age at treatment and the root filling extravasation.

## Introduction

The ideal root canal filling material for a pulpectomy (PE) in primary teeth should be antimicrobial, easy to manipulate, easily removed, resorbable, biocompatible, cost effective, radiopaque, and should not harm the periapical tissues or affect the development or eruption of the permanent teeth. 1 2 3 4 Traditionally, zinc oxide eugenol (ZOE) has been the material of choice for PE. The success rate of ZOE PE ranges from 74.5 to 100%. 5 6 7 8 9 10 However, several studies found that ZOE resisted resorption and might deflect the permanent tooth eruption path. 2 5 11 12

The American Academy of Pediatric Dentistry recommends using a resorbable material, such as nonreinforced ZOE, iodoform-based paste, or a combination paste of iodoform and calcium hydroxide to fill the canals. 13 Premixed calcium hydroxide and iodoform paste (Vitapex; Neo Dental Chemical Product Co., Ltd, Tokyo, Japan) is commonly used due to its excellent clinical and radiographic outcomes. 6 7 14 Many studies reported favorable results of Vitapex PE in primary teeth, ranging from 80 to 100%. 6 7 8 14 15 16 The main advantage of Vitapex is its resorbability. When extruded from the root apex, Vitapex resorbs beginning at 1 week to 3 months without causing a foreign body reaction. 7 14 17 18

The follow-up periods in most clinical studies evaluating the Vitapex PE success rate ranged from 3 to 18 months. 6 7 8 10 14 15 16 19 20 21 22 The factors associated with the PE success rate can be used to predict the treatment outcome. Several ZOE studies found factors that were significantly related to PE success in primary teeth. 5 23 24 25 26 However, few studies have focused on the factors influencing the success of Vitapex PE in primary teeth. 10 21 22 Some authors observed that early resorption of Vitapex in the root canals created voids in the canal, leading to the formation of a hollow tube. 6 7 17 27 This tube allows bacterial reinfection and leads to Vitapex resorption. Excessive resorption of a root canal filling affects the PE success rate. 10 28 Therefore, our aims were to evaluate the success rate of Vitapex PE, the Vitapex resorption rate, and their related factors in primary teeth.

## Materials and Methods

This retrospective study was approved by the Institutional Review Board at the Faculty of Dentistry/Faculty of Pharmacy, Mahidol University, Bangkok, Thailand (COA.No.MU-DT/PY-IRB2019/007.1601). Patient dental records from the Pediatric Dental Clinic at the Faculty of Dentistry, Mahidol University from 2013 to 2019 were used in this study. The data were recorded if the information met the inclusion criteria: pulpectomized teeth using Vitapex (Neo Dental Chemical Product Co., Ltd, Tokyo, Japan) as the obturating material and included symptoms, clinical examination, preoperative, immediate, and postoperative digital radiographs; and a follow-up period of at least 6 months. Medically compromised children were excluded from the analysis. The sample size calculation was based on a previous study 8 that reported an 89% success rate of Vitapex PE, with a 5% margin error. The power of the study was set at 95% with α = 0.05 as the statistical significance level. Therefore, at least 404 Vitapex PE teeth were required.

The clinical, radiographic, and intervention procedures and related information was recorded. The characteristics of each patient, that is, age at treatment and tooth type, number of visits, intracanal medication, behavior, operator skill, and time to final restoration, were recorded from the dental charts. Preoperative radiographic findings, preoperative root resorption, obturation quality, and Vitapex resorption were evaluated using digital periapical radiographs.

The preoperative radiographs were used to determine the preoperative radiographic findings and preoperative root resorption. The preoperative radiographic findings were categorized as no pathology, discontinuous lamina dura/widened periodontal space (PDS), furcation involvement, and periapical lesion. 8 29 No pathology was defined as no pathologic change in the lamina dura and/or PDS at the furcation and/or the root length. A discontinuous lamina dura and/or widened PDS were defined as a minimal change in the lamina dura or PDS at the furcation and/or along the root length. Furcation involvement was defined as a radiolucency at the furcation area, and a periapical lesion was defined as a radiolucency that extended from the furcation to the periapical area. Preoperative root resorption was classified as the absence or presence of preoperative root resorption. 5

The immediate postoperative radiographs were used to determine the obturation quality. 10 30 Adequate root filling was defined as when Vitapex reached the apex or was 1 to 2 mm short of the apex. Short filling was defined as when the Vitapex filling was more than 2 mm short of the radiographic apex, and Vitapex extruding beyond the root apex was considered extruded filling.

The treatment outcome and Vitapex resorption were assessed using postoperative radiographs at a follow-up of at least 6 months. PE was considered a failure when the radiograph demonstrated a progressive pathologic change with/without clinical signs or symptoms. 8 28 Vitapex resorption was defined as resorption when the material was resorbed more than 2 mm from the root apex. 10

Radiographic evaluation standardization was performed by two examiners evaluating 15% of the samples. The intraexaminer and interexaminer reliability is acceptable when the kappa values are more than 0.8. 31 The interexaminer reliability kappa value of the two examiners was 0.85, and the intra-examiner reliability was 0.9 and 0.87, respectively.

The data were analyzed using the statistical package for social sciences version 28.0 and expressed as frequencies and percentages based on the independent variables. The Kaplan–Meier method was used to estimate the success rate of the Vitapex PE and Vitapex resorption rate. The study endpoint was set at a 60-month follow-up. A Vitapex PE that demonstrated no signs of failure or natural exfoliation or lost to follow-up was considered a censored case. The end of the observation time of each censored case was the last follow-up date in the dental record. The observation time of the cases that had no failure event until the end of the study was defined as 60 months. Cox regression analysis was used to identify the failure factors of Vitapex PE and factors related to Vitapex resorption. The significance level was set at *p <* 0.05.

## Results

A review of 3,246 dental records found 448 patients (252 males and 196 females) with 647 Vitapex PEs that met the inclusion criteria. There were 93 upper primary anterior teeth and 554 primary posterior teeth at the time of treatment, and the patient's ages ranged from 19 to 121 months (mean age = 63.8 ± 19.7 months).

**Fig. 1:**
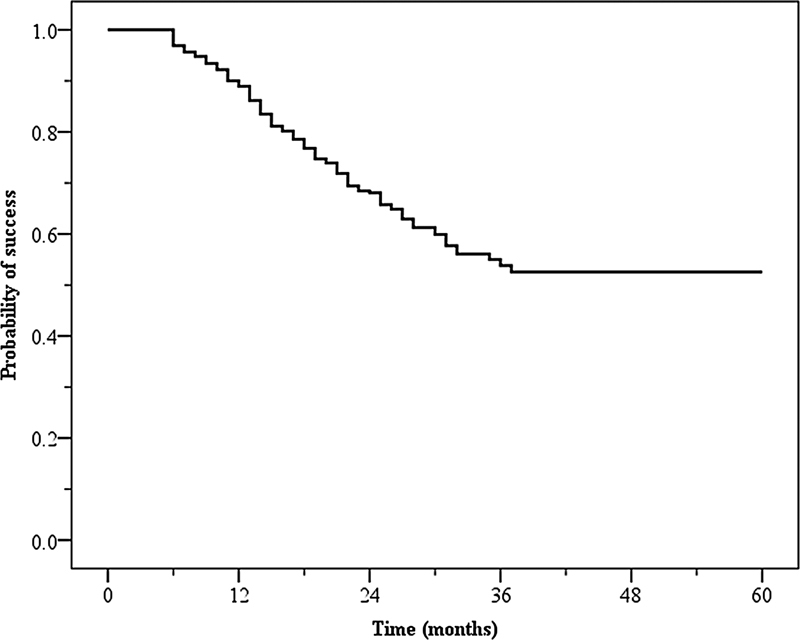
Probability of Vitapex pulpectomy success.

The probability of Vitapex PE success at the 60-month follow-up is presented in Fig. 1 . The Vitapex PE success rate was 88.9 and 68.1% at the 12- and 24-month follow-up, respectively. However, at the 36 to 60-month follow-ups, the success rate remained stable at 53.8%. The factors that affected Vitapex PE failure are presented in Table 1 . The univariable cox regression analysis demonstrated that age, preoperative radiographic findings, obturation quality, and Vitapex resorption were significantly associated with the Vitapex PE failure rate. However, Vitapex PE failure was only significantly related to age and preoperative radiographic findings in the multivariable cox regression analysis. Patients 36 to 72-month-old ( *p* = 0.01) and >72-month-old ( *p* < 0.01) demonstrated significantly more Vitapex PE failures compared with patients less than 36-month-old. For the preoperative radiographic factors, a periapical lesion ( *p* < 0.01) and furcation involvement ( *p* < 0.01) significantly increased PE failure compared with no pathology. In contrast, a discontinuous lamina dura/widened PDS did not significantly affect PE success compared with no pathology.

**Table 1:** Cox regression analysis of factors related to Vitapex PE failure

Factors	*n*	Failures (%)	Univariable analysis		Multivariable analysis	
			HR (95% CI)	*p* -Value	Adjusted HR (95% CI)	*p* -Value
1. Age (months)						
< 36	49	4 (8.16)	1		1	
36–72	402	115 (28.6)	3.63 (1.34, 9.84)	0.01 a	3.70 (1.34, 10.23)	0.01 a
> 72	196	58 (29.6)	4.77 (1.73, 13.15)	<0.01 a	4.72 (1.67, 13.35)	<0.01 a
2. Tooth type						
Primary anterior	93	21 (22.58)	1		–	–
Primary first molar	225	67 (29.78)	1.51 (0.93, 2.47)	0.10		
Primary second molar	329	89 (27.05)	1.34 (0.83, 2.15)	0.23		
3. Preoperative radiographic findings						
No pathology	94	10 (10.64)	1		1	
Discontinuous lamina dura/ widened PDS	259	62 (23.94)	2.25 (1.15, 4.38)	0.02 a	1.92 (0.98, 3.75)	0.06
Furcation involvement	221	74 (33.48)	3.11 (1.61, 6.03)	<0.01 a	2.62 (1.35, 5.08)	<0.01 a
Periapical lesion	73	31 (42.47)	4.48 (2.19, 9.13)	<0.01 a	4.77 (2.32, 9.82)	<0.01 a
4. Preoperative root resorption						
Absence	636	173 (27.20)	1		–	–
Presence	11	4 (36.36)	1.20 (0.44, 3.22)	0.73		
5. Number of treatment visits						
One visit	292	72 (24.66)	1		–	–
≥Two visits	355	105 (29.58)	1.16 (0.86, 1.57)	0.32		
6. Intracanal medication						
Formocresol	59	17 (28.81)	1		–	–
Calcium hydroxide	296	88 (29.73)	1.21 (0.72, 2.03)	0.48		
None	292	72 (24.66)	1.00 (0.59, 1.70)	1.00		
7. Quality of obturation						
Adequate	332	77 (23.19)	1		1	
Extruded	235	75 (31.91)	1.46 (1.06, 2.01)	0.02 a	1.28 (0.92, 1.79)	0.14
Short	80	25 (31.25)	1.39 (0.88, 2.18)	0.16	1.43 (0.91, 2.26)	0.12
8. Behavior						
Uncooperative	43	8 (18.60)	1		–	–
Potentially cooperative	310	88 (28.39)	1.61 (0.78, 3.32)	0.20		
Cooperative	294	81 (27.55)	1.73 (0.84, 3.58)	0.14		
9. Operator skill						
Specialist	67	16 (23.88)	1		–	–
Postgraduate student	520	141 (27.12)	1.13 (0.68, 1.90)	0.64		
Undergraduate student	60	20 (33.33)	1.51 (0.78, 2.91)	0.22		
10. Time to the final restoration						
Immediate	282	66 (23.40)	1		–	–
Intermediate	365	111 (30.41)	1.35 (0.99, 1.83)	0.06		
11. Vitapex resorption						
No	111	15 (13.51)	1		1	
≤1/2 of the root length	273	55 (20.15)	1.17 (0.66, 2.07)	0.60	1.07 (0.59, 1.92)	0.83
> 1/2 of the root length	263	107 (40.68)	1.78 (1.03, 3.06)	0.04 a	1.57 (0.90, 2.76)	0.12

Abbreviations: HR, hazard ratio; 95% CI, 95 percent confidence interval.

a The significance level was *p* < 0.05.

**Fig. 2:**
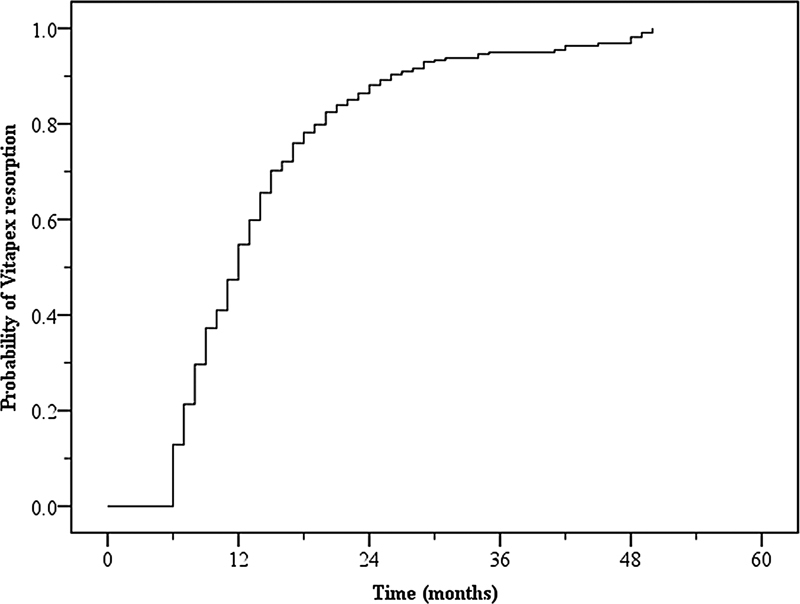
Probability of Vitapex resorption.

The Vitapex resorption rate is shown in Fig. 2 . Vitapex resorption increased over time and more than 50% of the pulpectomized teeth presented Vitapex resorption faster than physiologic root resorption at the 12-month follow-up. The factors that affected Vitapex resorption are presented in Table 2 . The Vitapex resorption was significantly related to age and obturation quality. Patients 36 to 72 month old and >72 month old demonstrated a 50% ( *p* = 0.02) and 63% ( *p* = 0.01), respectively, likelihood of Vitapex resorption, which was greater compared with patients <36 month old. Vitapex extruded beyond the apex had a 76% ( *p* < 0.01) greater likelihood of Vitapex resorption compared with a short root filling. In contrast, adequate root filling did not demonstrate a significantly different Vitapex resorption compared with short root filling.

**Table 2:** Cox regression analysis of factors related to Vitapex resorption

Factors	*n*	Resorption (%)	Median time (mo)	Univariable analysis		Multivariable analysis	
				HR (95% CI)	*p* -Value	Adjusted HR (95% CI)	*p* -Value
1. Age (months)							
< 36	49	36 (73.47)	13	1		1	
36–72	402	329 (81.84)	12	1.38 (0.98, 1.95)	0.07	1.50 (1.06, 2.12)	0.02 a
> 72	196	171 (87.24)	12	1.51 (1.51, 2.16)	0.03 a	1.63 (1.13, 2.35)	0.01 a
2. Tooth type							
Primary anterior	93	77 (82.80)	11	1		–	–
Primary first molar	225	196 (87.11)	12	1.13 (0.87, 1.48)	0.36		
Primary second molar	329	263 (79.94)	12	1.03 (0.80, 1.33)	0.81		
3. Preoperative radiographic findings							
No pathology	94	77 (81.91)	12	1		–	–
Discontinuous lamina dura/widened PDS	259	214 (82.63)	12	1.02 (0.79, 1.32)	0.88		
Furcation involvement	221	182 (82.35)	12	1.01 (0.77, 1.32)	0.95		
Periapical lesion	73	63 (86.30)	11	1.20 (0.86, 1.68)	0.28		
4. Preoperative root resorption							
Absence	636	527 (82.86)	12	1		–	–
Presence	11	9 (81.82)	6	1.69 (0.87, 3.26)	0.12		
5. Number of treatment visits							
≥Two visits	355	296 (83.38)	12	1		–	–
One visit	292	240 (82.19)	12	1.05 (0.88, 1.24)	0.58		
6. Intracanal medication							
Formocresol	59	56 (94.92)	12	1		–	–
Calcium hydroxide	296	240 (81.08)	12	1.11 (0.82, 1.48)	0.51		
None	292	240 (82.19)	12	1.14 (0.85, 1.53)	0.39		
7. Quality of obturation							
Short	80	57 (71.25)	14	1		1	
Adequate	332	250 (75.30)	12	1.12 (0.84, 1.50)	0.43	1.16 (0.87, 1.55)	0.32
Extruded	235	229 (97.45)	10	1.68 (1.25, 2.24)	<0.01 a	1.76 (1.31, 2.35)	<0.01 a

Abbreviations: mo, months; HR, hazard ratio; 95%CI, 95 percent confidence interval.

a The significance level was *p* < 0.05.

## Discussion

This study used the Kaplan–Meier method to evaluate the success probability of Vitapex PE with 60-month follow-up periods. The Vitapex PE success rate was 96.9, 88.9, and 76.8% at the 6-, 12-, and 18-month follow-up, respectively. The success rate of Vitapex PE decreased over time. Our results are similar to those of most previous studies that reported Vitapex PE success rates ranging from 78 to 100% with follow-up periods of 3 to 18 months. 6 7 8 14 15 21 22 However, some studies demonstrated a lower success rate of Vitapex PE than our findings. 10 20 Chen et al 10 found a Vitapex PE success rate of 94.5, 60.7, and 53.6% at the 6-, 12-, and 18-month follow-up, respectively. These results indicated a high rate of Vitapex resorption that is faster than root resorption, resulting in Vitapex PE failure. Another study reported a Vitapex PE success rate of 80% at the 6-month and 56% at the 12-month follow-up, the lower success rate was due to the sample selection where treatment was performed in teeth with a poor prognosis. 20

Vitapex PE failure was significantly related to the patient's age and preoperative radiographic findings. In contrast, tooth type, preoperative root resorption, number of treatment visits, intracanal medication, obturation quality, behavior, operator skill, time to the final restoration, and Vitapex resorption were not significantly associated with Vitapex PE failure.

This study found that younger patients had a significantly higher success of Vitapex PE compared with older patients, which agreed with a previous study. 21 This might be because the root canal anatomy changes with age due to secondary dentin deposition that reduces the root canal diameter or leads to canal calcification, increasing the difficulty of root canal preparation, cleansing, and shaping the root canal. 29 32 Less dentin deposition in the younger child's root canal and being easier to debride resulted in a greater chance of success.

For the preoperative radiographic finding, teeth without pathology had a significantly higher success rate than teeth with a periapical lesion or furcation involvement. However, teeth with a discontinuous lamina dura/widened PDS demonstrated similar success compared with teeth without pathology. This finding coincides with several studies 24 33 34 and can be used to predict the treatment outcome of Vitapex PE. The severity of the radiographic change indicates the progression of infection in time and extent. An extensive lesion might impede periradicular tissue healing. 29 Therefore, teeth with a large preoperative lesion had a lower chance of success compared with teeth with a small lesion.

The time until the final restoration was not related to the Vitapex PE success rate. Immediately restored teeth that received a final restoration after filling the root did not demonstrate a significant difference in success compared with teeth with an intermediate restoration. These results contrasted with those of a previous study that found using an intermediate restoration was associated with PE failure. 22 These disparate findings might be because the intermediate restorations in the present study had adequate coronal seals.

Moreover, our study demonstrated that teeth with Vitapex resorption>1/2 of the root length had 1.57-fold greater likelihood of failure compared with teeth without Vitapex resorption. However, the Vitapex resorption was not related to the PE success rate, which was different from a previous study. 10 The study found that an excessive resorption rate of Vitapex reduced both clinical and radiographic success rates. Vitapex resorption can create voids and bacteria reinfection in the root canal, known as a hollow tube. 6 27 However, Vitapex resorption did not influence the Vitapex PE outcome. Our study found a high rate of Vitapex resorption in which 50% of the cases were found at the 12-month follow-up, especially Vitapex extravasation cases. Identifying the factors related to Vitapex resorption can be beneficial for avoiding a hollow tube.

These results demonstrated that the patient's age and obturation quality were significantly related to Vitapex resorption. Our results indicated that patients 36 to 72-month-old and >72-month-old had a higher probability of Vitapex resorption compared with younger patients. The reason for this might be that primary root resorption begins at the site of the primary tooth root that is closest to the permanent tooth and induces osteoclasts to resorb the root and root filling material. 35 Therefore, older patients have a greater chance of resorption compared with younger patients due to more physiologic root resorption and having less distance between the primary tooth root and permanent tooth bud. Another factor related to Vitapex resorption was Vitapex extruded beyond the root apex. This finding might be because the extravasation of Vitapex induces macrophages to resorb the excess filling and causes overproduction of macrophages to resorb the extruded and/or intracanal filling material. 17 Moreover, the present study found that the prevalence of extruded Vitapex was 6.4, 63.4, and 30.2% in the <36-, 36 to 72-, and >72-month-old patients, respectively.

The survival of Vitapex PE teeth determines the probability that a tooth will be retained in the oral cavity after treatment. Tooth survival outcome is important information that should be discussed with the caregiver before treatment. In this study, factors related to the success rate of Vitapex PE were the patient's age and preoperative lesion. Therefore, these factors should be used to predict the outcome of Vitapex PE.

The present study has several limitations. Because this was a retrospective study, some variables, such as multiple operators and various follow-up times among subjects, could not be controlled. Therefore, the survival analysis and Cox regression analysis were used to calculate the success rate of the Vitapex PE and the Vitapex resorption rate, as well as the factors related to their success and resorption so that all the data could be taken into account. Although Vitapex PE was performed by numerous operators with a variety of clinical experience, the Cox regression analysis indicated that this was not significantly associated with Vitapex PE failure. However, the study also has some strengths, that is, a large sample size, and long follow-up period.

## Conclusions

1. The success rate of Vitapex PE decreased in the first 3 years of follow-up and was stable at 36 to 60 months. The patient' age and preoperative radiographic findings influenced PE failure.
2. More than 50% of the PE teeth demonstrated Vitapex resorption by the 12-month follow-up. Vitapex PE in older patients and extravasation of Vitapex beyond the root apex resulted in higher Vitapex resorption.
